# A Multimodal, SU-8 - Platinum - Polyimide Microelectrode Array for Chronic *In Vivo* Neurophysiology

**DOI:** 10.1371/journal.pone.0145307

**Published:** 2015-12-18

**Authors:** Gergely Márton, Gábor Orbán, Marcell Kiss, Richárd Fiáth, Anita Pongrácz, István Ulbert

**Affiliations:** 1 Institute of Cognitive Neuroscience and Psychology, Research Centre for Natural Sciences, Hungarian Academy of Sciences, Magyar tudósok körútja 2, building Q2, H-1117, Budapest, Hungary; 2 Department of Microtechnology, Institute for Technical Physics and Materials Science, Centre for Energy Research, Hungarian Academy of Sciences, Konkoly Thege M. út. 29–33, H-1121, Budapest, Hungary; 3 School of Ph.D. Studies, Semmelweis University, Ü llői út 26, H – 1085, Budapest, Hungary; 4 Department of Electron Devices, Budapest University of Technology and Economics, Magyar tudósok körútja 2, building Q, H-1117, Budapest, Hungary; 5 Faculty of Information Technology and Bionics, Pázmány Péter Catholic University, Práter utca 50/a, H-1083, Budapest, Hungary; Consejo Superior de Investigaciones Cientificas - Instituto Cajal, SPAIN

## Abstract

Utilization of polymers as insulator and bulk materials of microelectrode arrays (MEAs) makes the realization of flexible, biocompatible sensors possible, which are suitable for various neurophysiological experiments such as *in vivo* detection of local field potential changes on the surface of the neocortex or unit activities within the brain tissue. In this paper the microfabrication of a novel, all-flexible, polymer-based MEA is presented. The device consists of a three dimensional sensor configuration with an implantable depth electrode array and brain surface electrodes, allowing the recording of electrocorticographic (ECoG) signals with laminar ones, simultaneously. *In vivo* recordings were performed in anesthetized rat brain to test the functionality of the device under both acute and chronic conditions. The ECoG electrodes recorded slow-wave thalamocortical oscillations, while the implanted component provided high quality depth recordings. The implants remained viable for detecting action potentials of individual neurons for at least 15 weeks.

## Introduction

In the last few decades, the range of experimental neuroscience methods has been extremely widened by various technological advances. A remarkable segment of this progress was fueled by the utilization of microelectromechanical systems (MEMS) technology for the fabrication of high density microelectrode arrays (MEAs). Following the appearance of the first silicon-based micromachined neural implants [1], such devices evolved rapidly and today a great variety of precisely and reproducibly fabricated MEAs are available, which make the recording of potential changes in the extracellular space with high spatial density possible [2–5].

The biocompatibility of the MEAs is crucial, especially if the devices are intended to be in contact with the tissue on the long term. Typical MEMS materials, such as Si, SiO_2_, Si_3_N_4_ and metals such as gold, platinum and iridium are non-toxic and inert [6–8]. However, in terms of biocompatibility, these are only necessary, but not sufficient conditions, since inert materials can also trigger the foreign body response of the immune system and cause glial scar formation, which can compromise the functionality of the electrodes [9]. A huge advantage of polymer-based depth MEAs is their mechanical flexibility, which allows smoother coupling with the soft tissue than rigid materials [10]. A flexible neural implant can follow small motions and pulsations of the brain, therefore causes less disturbance in its environment. Several biocompatible polymers, e.g. SU-8 photoresist [11], Polyimide (PI) [12] and Parylene C [13, 14] can be employed as bulk and insulator materials of neural sensors.

The palette of polymer-based MEAs utilized in neurophysiology is diverse. It contains devices developed for interfacing both with the peripheral and with the central nervous system. Peripheral neurons can be contacted with either implants penetrating into the nerves [15, 16] or cuff electrodes, which can be wrapped around them [17]. Such devices can serve as key elements of brain-machine interfaces (BMIs). Similarly, flexible retinal implants are used for vision restoration for patients suffering from retinitis pigmentosa [18]. Polymer-based probes which are implantable into the central nervous system have also been realized [19–22], including double-sided electrode arrays [23] and probes with drug delivery capabilities [24, 25].

Flexible devices are also frequently utilized for electrocorticography (ECoG), a method that uses electrodes placed directly on the exposed surface of the brain [26]. In the clinics, ECoG is widely used during treatment of patients suffering from epilepsy, whose condition necessitates surgical resection [27–29]. Such surgeries require precise localization of the epileptogenic zones. Due to its higher spatial resolution and signal-to-noise ratio (SNR) compared to electroencephalography, ECoG is more suitable for this purpose [30]. The technique is employed not only to assess the location of the irritative zones from ictal spike and interictal epileptiform activity, but also for functional mapping to avoid causing damage to critical regions. In neurosciences, ECoG can be used for functional mapping of various cortical regions, e.g. the vibrissa/barrel field of rat neocortex [31]. For such purposes, a variety of ECoG electrode arrays have been fabricated of polymers such as polydimethylsiloxane (PDMS), Parylene C and polyimide [32–34].

In spite of the wide range of flexible, polymer-based neural sensors, most of them are developed for a single type of measurement. In this paper we report the fabrication and functional characterization of a multimodal MEA, consisting of an ECoG part (with 8 electrodes) and a single-shank, implantable part (with 16 electrodes), allowing simultaneous surface and depth recordings.

## Materials and Methods

### 2.1. Design concepts

**Fig 1:**
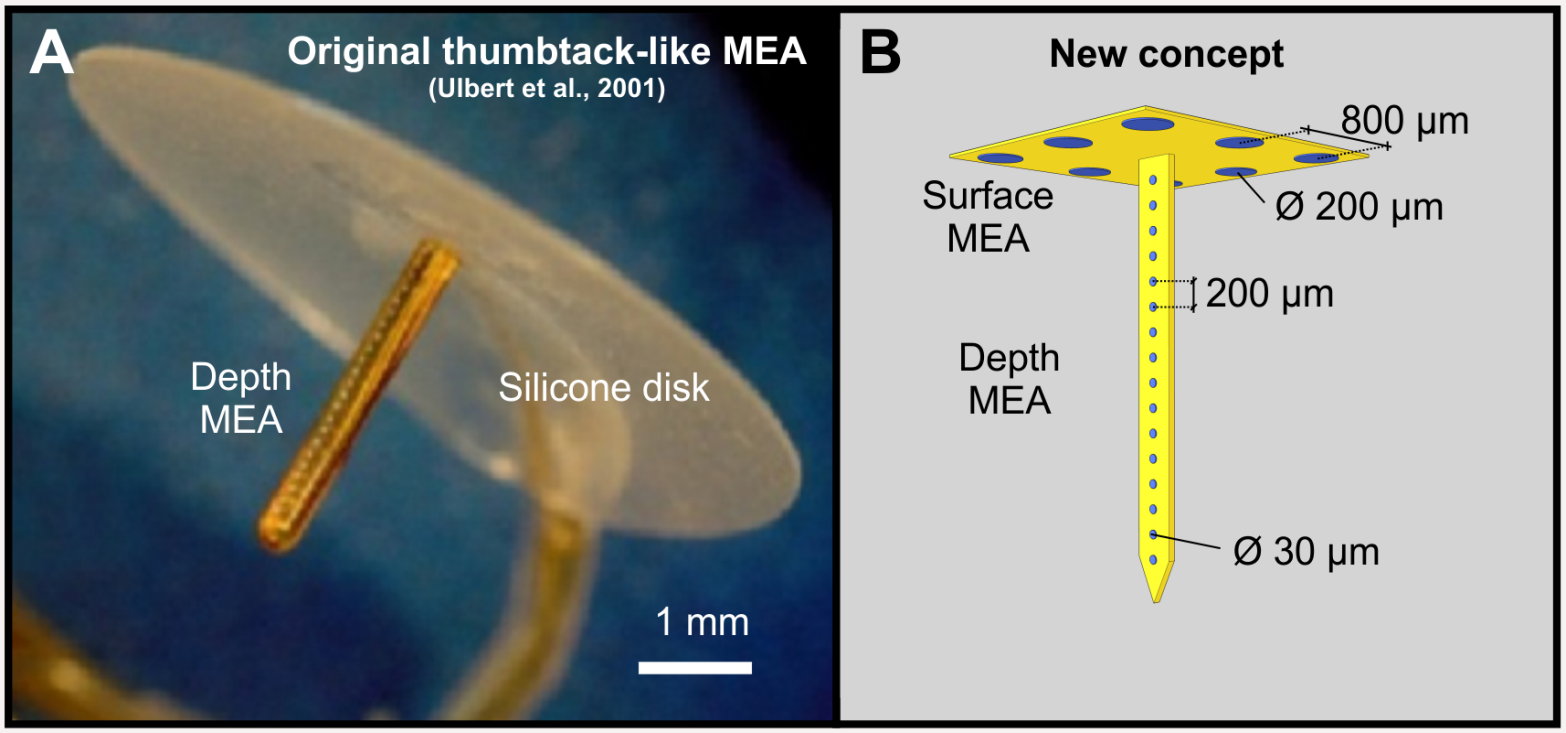
Design concept. (A) The thumbtack electrode, containing a 3 mm long shaft with an array of insulated fine wires and a silicone disk (www.plexon.com). (B) Design concept of the multimodal polymer-based MEMS electrode array.

The probe is an upgraded version of the thumbtack-like neural MEA, shown in Fig 1A, which had been successfully used for recording field potentials, multiple unit and single unit activities in behaving and anaesthetized humans [35]. The thumbtack-like sensor contains a laminar array of polyimide isolated platinum–iridium electrodes on a single shaft with an outer diameter of 350 μm and length of 3 mm. The shaft can be implanted into the cerebral cortex perpendicularly to the surface of the brain. It protrudes perpendicularly out of the center of an 8 mm diameter silicone disk. The disk facilitates the immobility of the shaft during recordings by plying to the brain surface. We intended to modify this device by equipping the disk with an electrode array as well, thus enabling ECoG recordings in the vicinity of the implanted shaft. At the same time, we substituted the hand-made shaft with a polymer-based MEMS component in order to achieve a more precise and reproducible fabrication and fine mechanical coupling between the probe and the tissue. The ECoG component was designed to be equipped with eight relatively large circular sites (d = 200 μm) for field potential recordings. The depth MEAs were designed to contain smaller electrodes (d = 30 μm), which might be suitable for the detection of action potentials of individual neurons within the tissue.

### 2.2. Fabrication methods

In this chapter we present the process flow used for the realization of the microfabricated components. The rapid and cost-effective procedure had already been successfully employed for the construction of a linear array of electrodes, used as an ECoG [36]. The processes resulted in a PI (bottom insulator)–TiO_x_/Pt (conductive)–SU-8 (top insulator) layer structure. The implantable MEA and the ECoG component were fabricated with the very same MEMS processes. Their layouts were merged onto a joint wafer layout in order to reduce the number of photolithographic masks needed. In the final form of the device, the TiOx/Pt conductive layer is employed for multiple purposes: it functions as electrode contact sites, wiring and connector pads. The bottom insulator layer (PI) provides electrical insulation for the bottom side of the probe everywhere, while the top insulator layer (SU-8) is opened above the electrode contact sites and connector pads and only insulates the leads. We have found this polymer composition beneficial in a couple of aspects. The adhesion of PI on SiO2 was sufficient for both enduring the fabrication steps and for ensuring easy sample removal. Since SU-8 is a commonly used negative photoresist, its patterning is more straightforward, which makes it suitable to form the top insulator layer.

**Fig 2:**
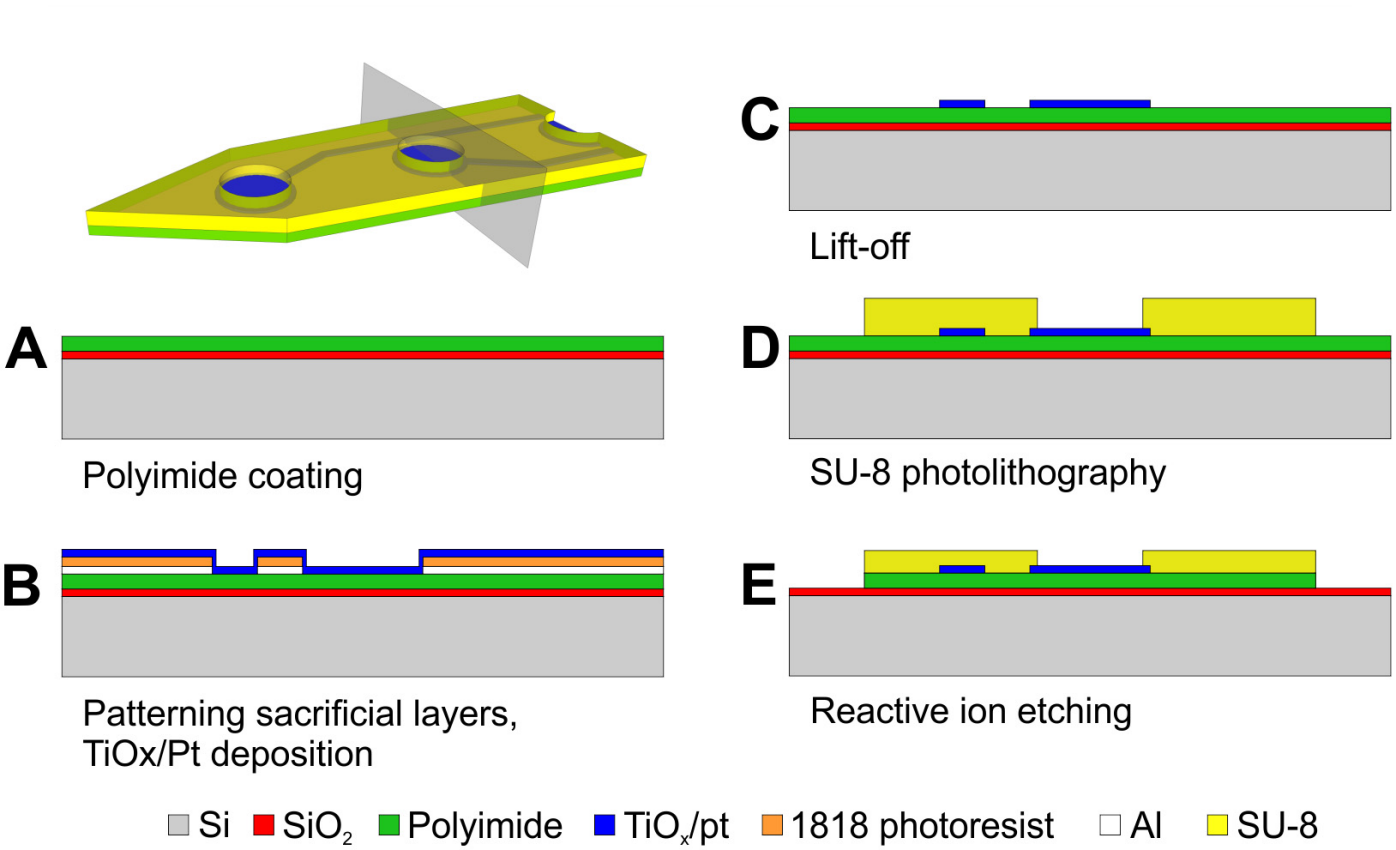
Steps of microfabrication. The processes yield a PI (bottom insulator)–TiO_x_/Pt (conductive)–SU-8 (top insulator) layer structure.

**Fig 3:**
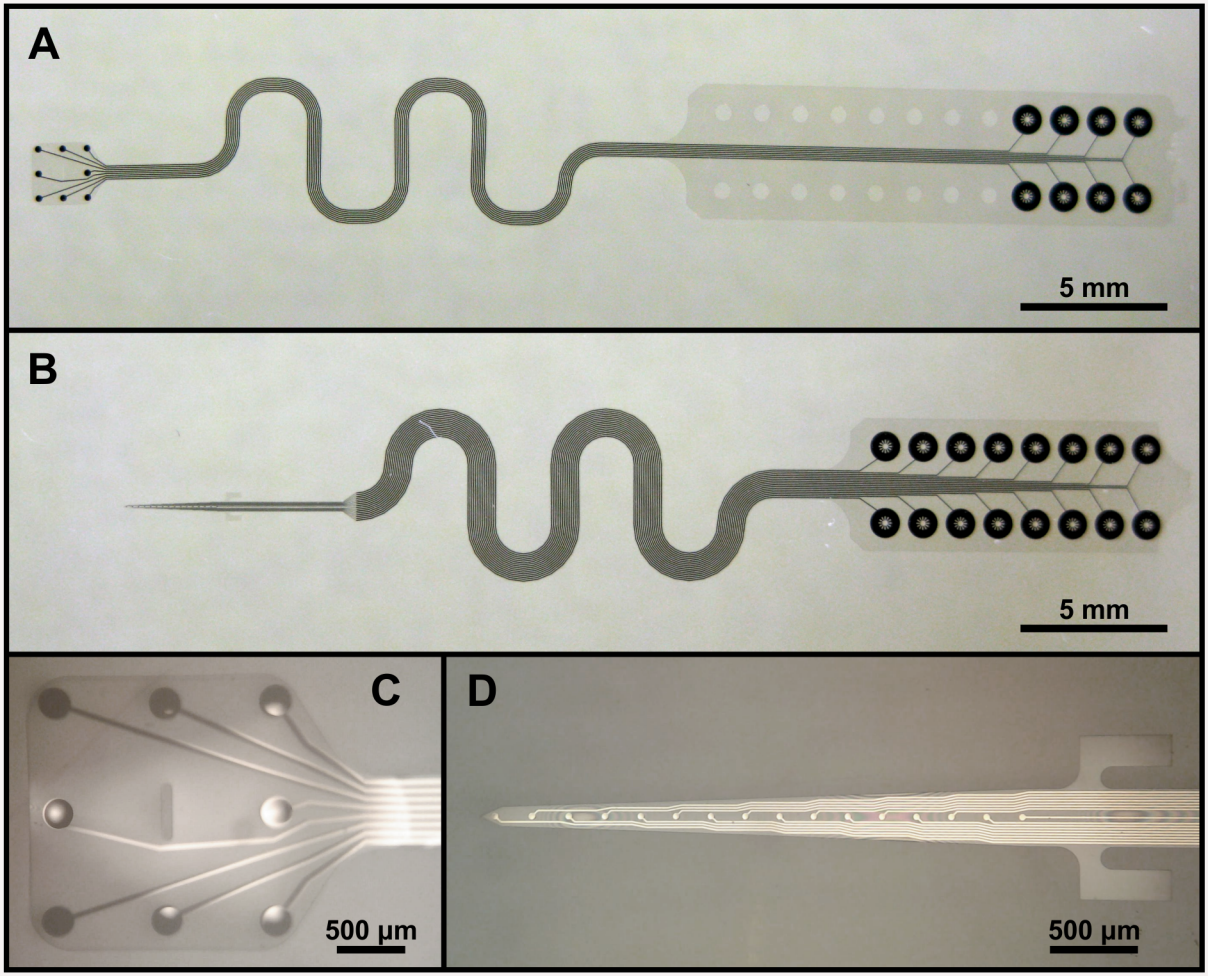
The microfabricated components. The surface MEA, shown in (A) and (C) is provided with a hole in the middle of the array, into which the depth MEA (shown in (B) and (D) can be inserted. Above the depth electrodes, two handles protrude from the sides of the shank.

Fig 2 shows the schematics of the main process steps. 4-inch silicon wafers were used as handling substrates of the polymer layers. First a 1 μm thick SiO_2_ layer was grown using wet oxidation at 1150°C. Following this, a 3.5 μm thick P84 polyimide layer (Evonik Industries, Essen, Germany) was spin-coated onto the front side of the wafer, as shown at step (A). An Al layer of 500 nm was deposited by evaporation, which was followed by the spin-coating of a 1.8 μm thick, Microposit 1818 (Dow Electronic Materials, Newark, DE, USA) photoresist. The resist was UV exposed, using a mask of 1 μm resolution. This pattern was transferred into the Al layer by wet chemical etching of the metal at room temperature, using a solution of H_2_O, CH_3_COOH, H_2_SO_4_, H_3_PO_4_, and HNO_3_ in a ratio of 70:20:30:32:20. 15 nm thick TiO_x_ layer was sputter-deposited for proper adhesion, followed by 270 nm of Pt, as shown at step (B). The rest of the photoresist and Al were etched away in acetone and in the solution mentioned before, respectively. The lift-off yielded a patterned layer of TiOx/Pt, which will function as the conductive material for the electrode sites, bonding pads and wirings (C). In the next step, a 20 μm thick SU-8 (by Micro Chem Corporation, Newton, MA, USA) layer was spin-coated and patterned with photolithography (D), during which the electrode sites and bonding pads were exposed and the contours of the microfabricated components of the devices were shaped. The process flow was continued with reactive ion etching (RIE) with a gas mixture of O_2_ and CF_4_ gases in a ratio of 1:1. In this step, the pattern of the SU-8 layer was transferred into the PI layer. While the exposed PI was etched completely, the SU-8 was only thinned to a thickness of approximately 12 μm. At the same time, Pt functioned as an etch-stop layer, protecting the PI below the future electrodes (S1 Fig shows thus created structures on a substrate wafer). Finally, the wafers were submerged into distilled water and the flexible MEMS structures were peeled off from the substrates with a pair of tweezers. The SiO_2_ layer underneath them remained on the Si wafer. Photographs of the two microfabricated components of the device are shown in Fig 3.

### 2.3. Assembly and packaging

**Fig 4:**
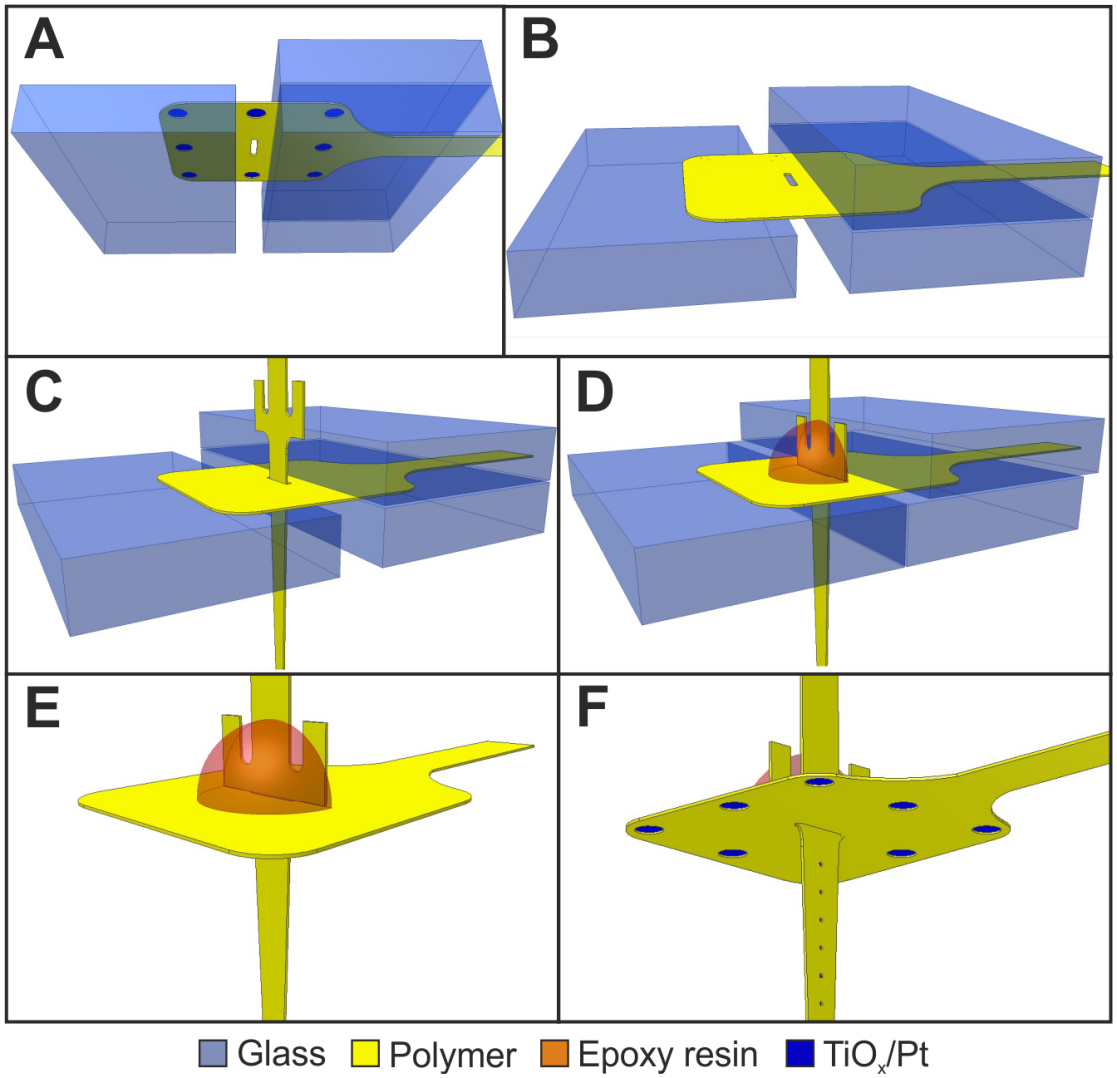
The assembly of the sensor components. (A) The surface MEA is clamped with glass slides. (B)-(C) The depth MEA is inserted and it is constrained by the lower glass slides. (D) The components are glued together with epoxy resin. (E) The glass slides are removed.

In order to assemble the device, we clamped the ECoG component between three pieces of 1 mm thick glass slides, as illustrated in Fig 4. At step (A), only the ECoG part was clamped with two slides from the bottom and one from the top so that the hole in the middle of its sensor region was not covered. The shank of the depth electrodes was inserted into the hole with a pair of tweezers (B). The shank was equipped with two handles, located on the sides, 300 μm above the electrode array. The insertion was complete when both of these handles mechanically contacted the ECoG component, and doing so they ensured the perpendicularity of the two components in one direction. Constraining the shaft of the depth MEAs with the bottom two glass slides provided perpendicularity in the other direction (C). The two components were fixed together with a drop of two-component epoxy resin, at their backsides, avoiding the electrodes (D). After one hour, the epoxy cured and the glass slides were removed (F). In the final step, the devices were equipped with connectors (Preci-Dip, Delémont, Switzerland). Their pins were stitched through the holes at the bonding pads of the microfabricated components and bonded onto exposed Pt sites, which had been formed in the vicinity of the holes with the same methodology as the electrodes.

### 2.4. Electrode impedance measurement and reduction methods

Characterization of the electrode impedances was performed by electrochemical impedance spectroscopy (EIS) in physiological saline (0.9% w/v of NaCl), employing an Ag/AgCl reference electrode (Radelkis Ltd., Hungary) and a platinum wire counter electrode with relatively high surface area. The probe signal was sinusoidal, with an RMS value of 25 mV. A Reference 600 instrument (Gamry Instruments, PA, USA) was used as a potentiostat and Gamry Framework 6.02 and Echem Analyst 6.02 software were used for experimental control, data collection and analysis. Experiments were performed in a Faraday cage.

In order to reduce the impedance of the depth electrodes, additional Pt was electrochemically deposited onto them. The Reference 600 instrument was used again, as a potentiostat. The electrochemical cell consisted of a solution of 1 g PtCl_4_ x 2HCl x 6 H_2_O + 2 cm^3^ conc. HCl + 200 cm^3^, an Ag/AgCl reference electrode and a Pt counter electrode. The deposition was performed for 10 minutes, on 100 mV (vs. reversible hydrogen electrode). The durability of such a platinized platinum (Pt/Pt) layer has been previously tested on silicon probes [37].

### 2.5. *In vivo* recording methods

#### 2.5.1. Acute tests

Electrophysiological recordings were performed in the rat brain in order to test the functionality of the MEAs. A total of 4 Wistar rats, weighing 270–400 g, have been anesthetized with a ketamine-xylazine solution and prepared for stereotaxic operation as described elsewhere [38]. Animals for both acute and chronic tests were kept and handled in accordance with the European Council Directive of 24 November 1986 (86/609/EEC), the Hungarian Animal Act, 1998 and the Animal Care Regulations of the Research Centre for Natural Sciences of the Hungarian Academy of Sciences (RCNS-HAS). The study was approved by the Institutional Animal Care and Use Committee of the RCNS-HAS (members: Dr. István Ulbert, Dr. József Topál and Péter Kottra). Animals had unlimited access to food and water, when they were awake. Each rat was kept in a 39 cm long, 22 cm wide, 18 cm high cage. They were under deep anesthesia during operations and recording sessions, as well as at the time of sacrifice. During anesthesia, paraffin oil was administered to their eyes to prevent them from drying. They were sacrificed by the injection of a lethal dose of ketamine/xylazine into the heart.

Craniotomy was performed -1.0 mm–-6.0 mm anteroposterior (AP), 2.0 mm–7.0 mm mediolateral (ML) in reference to the bregma. The implantation of the depth MEAs were targeted at the stereotaxic location of -3.36 mm AP, 5.5 mm ML, perpendicularly to the brain surface—which allowed laminar measurements in the barrel cortex and reaching into the hippocampus [39]. The dura mater was incised above the target location in order to achieve a smooth implantation. The probe was cramped with a curved, flat tip forceps by its depth MEA component, above the location where the depth MEA and the ECoG part had been joined. The forceps was forced to remain closed during implantation with a clamper and it was also rigidly connected to the moving arm of the stereotaxic apparatus. The arm allowed the manipulation of the probe with 10 μm precision in the dorsoventral and mediolateral directions and 100 μm precision in the anteroposterior direction. After the recordings, the probes were removed from the brain and cleaned. They were soaked in an aqueous solution of 10 mg/ml Terg-A-Zyme (by Alconox Inc., White Plains, NY, USA) for 10–15 minutes. 3–4 times during this period and after the probes were removed from the solution and rinsed with distilled water. After such cleaning process, no signs of organic residues were found on them.

Brain signal recordings were carried out using a 32-channel Intan RDH-2000 amplifier system (Intan Technologies LLC., Los Angeles, CA, USA) connected to a computer via USB 2.0, sampling with a frequency of 20 kHz. The reference electrode was a pointed stainless steel needle located beneath the skin posterior to the scalp. MATLAB 2014b (MathWorks Inc., Natick, MA, USA) and Edit 4.5 software of Neuroscan (Charlotte, NC, USA) was used for off-line signal visualization, filtering and analysis. Signals obtained by the depth MEA were subjected to CSD analysis. CSD was calculated with the MATLAB 2014b software (MathWorks Inc., Natick, MA, USA), with the utilization of the CSDplotter toolbox. For a clearer visualization, the CSD of 10 periods were averaged and plotted. The periods were aligned to each other based on the start of the upstates, i.e. the initiation of multiunit activity.

#### 2.5.2. Chronic recording capability tests

In order to characterize the recording capabilities of the MEA on the long term, two additional rats were successfully implanted chronically (in two other cases, the surgery was unsuccessful because of reasons not related to the device). These probes were inserted into the somatosensory (Rat-1) and motor (Rat-2) cortex. Until the implantation, the course of these operations was almost identical to the course of the acute tests, with the difference that screws were driven into the skull at the perimeter of the scalp opening. One of these screws served as a reference electrode. Following implantation, the craniotomy hole was filled with Gelaspon gelatin sponge (Germed, Rudolstadt, Germany). Dental acrylic cement (Vertex Pharmaceuticals, Boston, MA, USA) was used in order to cover the hole and in order to attach the electrical connector of the probe to the skull.

To avoid movement artifacts, the rats were anesthetized before chronic recordings with a mixture of 37.5 mg/ml ketamine and 5mg/ml xylazine at 0.2 ml/100 g. During each session, at least 10 minute long recordings were obtained with the same setup that was used for the acute tests. Later, 10 minute long sections of the signals were analyzed off-line for each session. The long-term stability of the surface electrodes was characterized by determining the amplitude spectral density of the signals measured with them. Average amplitude for frequencies corresponding to sleeping (below 4 Hz) was calculated. In case of depth electrodes, we focused on unit activity detection. in order to detect unit activities, the recording sections were band-pass filtered between 300 and 3000 Hz. The Klusters free software [40] was used for clustering (spike sorting), taking into account three principal components for each electrode. The clusters were manually accepted or discarded based on spike waveforms and autocorrelograms. Unit activities were only included in the analysis if the single unit signal-to-noise amplitude ratio (SU SNAR) of their clusters were higher than 2. Unit yield of the probe was determined as the total number of valid clusters on all of the 16 depth MEA channels. SU SNAR for each single unit clusters was calculated as follows.

$$SU\;SNAR_{i}\;=\;\frac{PP_{i}}{2\sigma_{n}}$$

where *i* is the index of the cluster, *n* is the index of the recording channel containing the spike waveforms of cluster *i*. *PP*_*i*_ is the mean peak-to-peak amplitude of the spikes (their corresponding 1.5 ms waveforms snippets) in cluster *i*, *σ*_*n*_ is the standard deviation of the filtered signal of the *n*th recording channel, of which its clustered unit activities are extracted.

## Results and Discussion

### 3.1. Microfabricated and assembled devices

**Fig 5:**
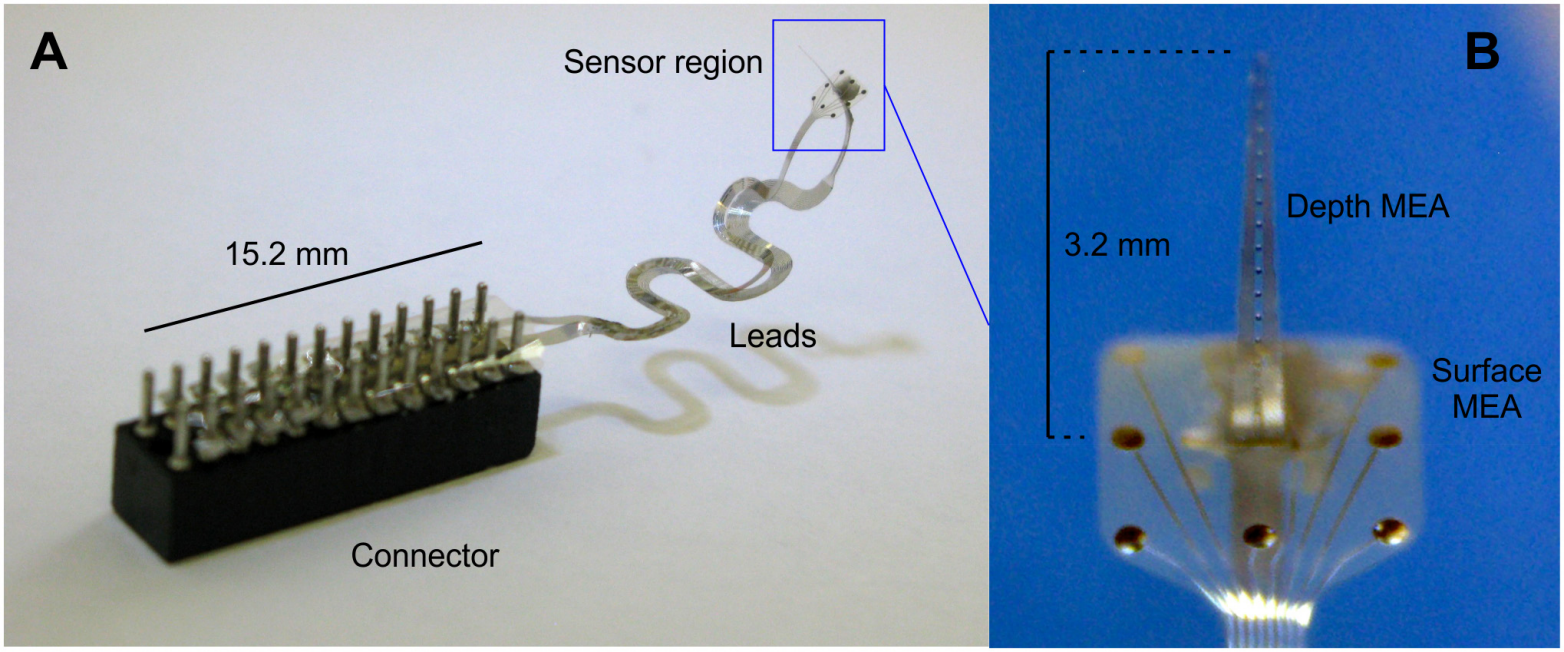
Photographs of the assembled device. (A) Macroscopic view. (B) A microscopic image of the sensor region, containing the microelectrodes.

An image of a device and a magnified view of its sensor region, containing the electrodes are presented in Fig 5. The microtechnological and assembly processes resulted in a probe geometry consistent with the design. The attachment of the shank (containing the depth electrodes) to the ECoG component was sufficient, the connection remained intact in all cases during the in vitro and in vivo tests. The similarly flexible meander transmission lines provided mechanical decoupling between the sensor region and the connector. The mechanical robustness of the lead was adequate, failures only occurred as a result of extreme pulling forces. Our overall experience was that these flexible tools do not require that much care during handling compared to the more brittle silicon-based depth MEAs.

### 3.2. Original and reduced electrode impedances in saline

**Fig 6:**
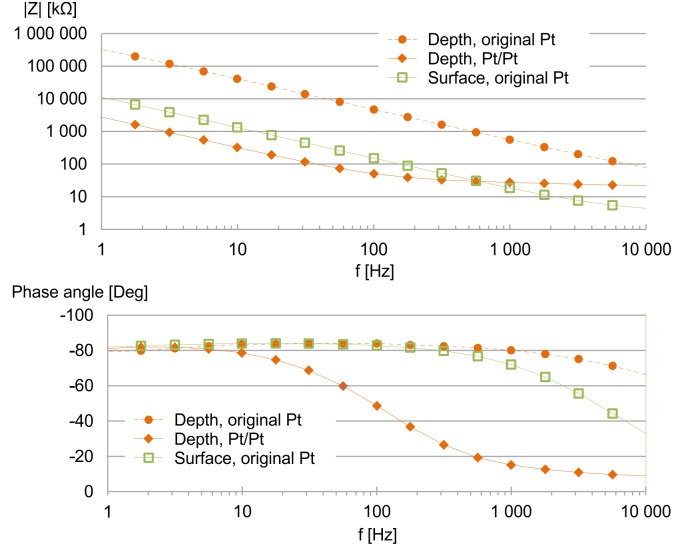
The average magnitude and phase values of electrode impedances at different frequencies, measured in physiological saline. The depth electrodes were subjected to electrochemical deposition of Pt, which reduced the impedance magnitudes from 559.5±148.4 kΩ to 27.6±8 kΩ at 1 kHz. The impedance of the surface sites were 18.6±0.5 kΩ on the average at 1 kHz, no modification was performed on them.

Results of average values yielded by in vitro impedance measurements on a probe are shown in Fig 6 (For exact results, see S1 Table). The original, sputtered thin-film Pt depth electrodes (with geometric area of 707 μm^2^) had an average impedance value of 559.5±148.4 kΩ at 1 kHz. We decided to reduce this value in order to obtain a better signal-to-noise ratio during measurements. The electrolytic deposition of platinum yielded a Pt/Pt layer of high roughness factor, hence the average impedance magnitude at 1 kHz reduced to 27.6±8 kΩ. As expected, the ECoG sites of larger (31400 μm^2^) geometric area had much lower original impedances: 18.6±0.5 kΩ on the average. Since ECoG electrodes are only expected to record local field potentials without unit activities, we found this value to be sufficient for this purpose and did not apply electrolytic deposition on these sites.

### 3.3. *In vivo* experimental results

**Fig 7:**
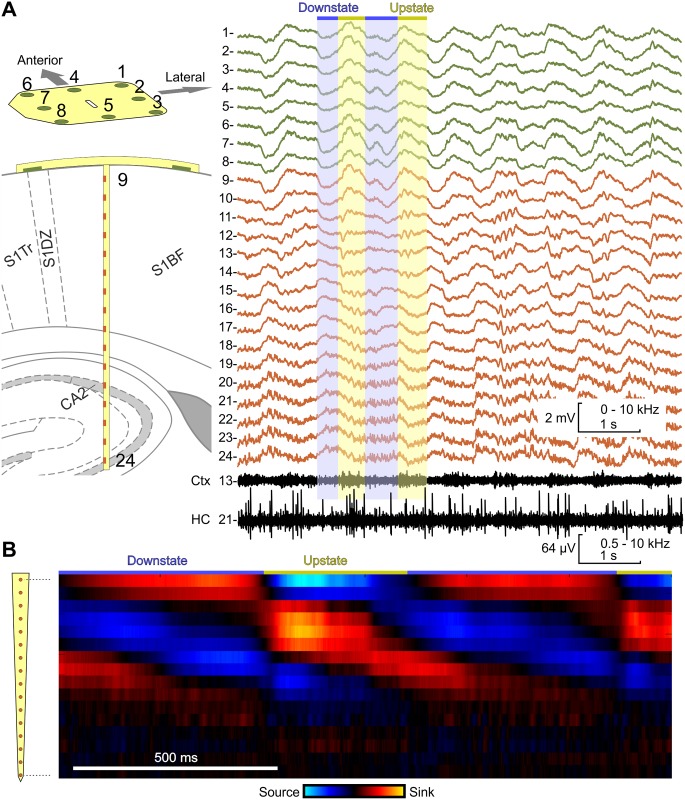
Acute *in vivo* tests. (A) Simultaneously recorded waveforms from the ECoG (channels no. 1–8, green curves) and the depth component (channels no. 9–24, red curves). The 1–1.5 Hz oscillation is the result of ketamine-xylazine anesthesia. During upstates, positive LFP can be detected on the brain surface and the upper cortical layers and negative LFP in deeper cortical layers. The intensity of unit activity (black curves) closely correlates with the upstate periods in the cortex, but not in the hippocampus. The brain atlas image has been reprinted from The rat brain in stereotaxic coordinates 2009, (ISBN 9780123742438), Paxinos et al ed, figure 61 under a CC BY license, with permission from Elsevier Ltd., original copyright 2009. (B) Changes of current sources and sinks in time, yielded from the signals recorded by the depth electrode array.

A representative sample of the recorded waveforms is presented in Fig 7 and in S1 Acute Data. Channels no. 1–8 represent local field potential (LFP) changes detected by ECoG electrodes. Channels no. 9–24 correspond to the depth electrode sites. A synchronous slow wave (1–1.5 Hz) oscillation can be observed on all channels, indicating slow-wave sleep (SWS), which is characteristic of the applied ketamine-xylazine anesthesia [41, 42]. Each period of the oscillation can be divided into two alternating states. Active (upstate) periods, when neuron membranes are depolarized and the cells generate action potentials (spikes) frequently, are followed by inactive (downstate) periods, when membranes are hyperpolarized and spikes do not occur [43, 44]. LFP has a positive peak during upstates on the brain surface and in the upper cortical layers, while in deeper layers the LFP polarity of the waves is reversed. This phenomenon can be observed on the ECoG channels and channels 9–18 of the implantable component. Elevated activity in higher frequency domains of the LFP signals on channels 19–24 indicate that the tip of the implanted shank reaches into the hippocampus, as expected. Unit activity was revealed by band pass filtering (500–5000 Hz). In the cortex, high intensity of multiunit activity can be observed within the upstate periods. In the hippocampus, unit activities do not follow the oscillation closely, which meets the expectations, since slow waves are supposedly generated by neocortical and thalamic oscillators [45].

The upstate phase of the oscillation begins with the formation of a current source in the upper and lower layers of the cortex and a massive sink in the middle layers. Entering the downstate phase, the CSD is transformed into a sink-source-sink pattern in the cortex, as shown in Fig 7B. This trend was also observed in humans during slow-wave sleep, although with different spatial pattern [46, 47]. The experiment suggests that in case the investigated neural tissue has a laminar structure, the probe makes the determination of current sinks and sources possible as well.

**Fig 8:**
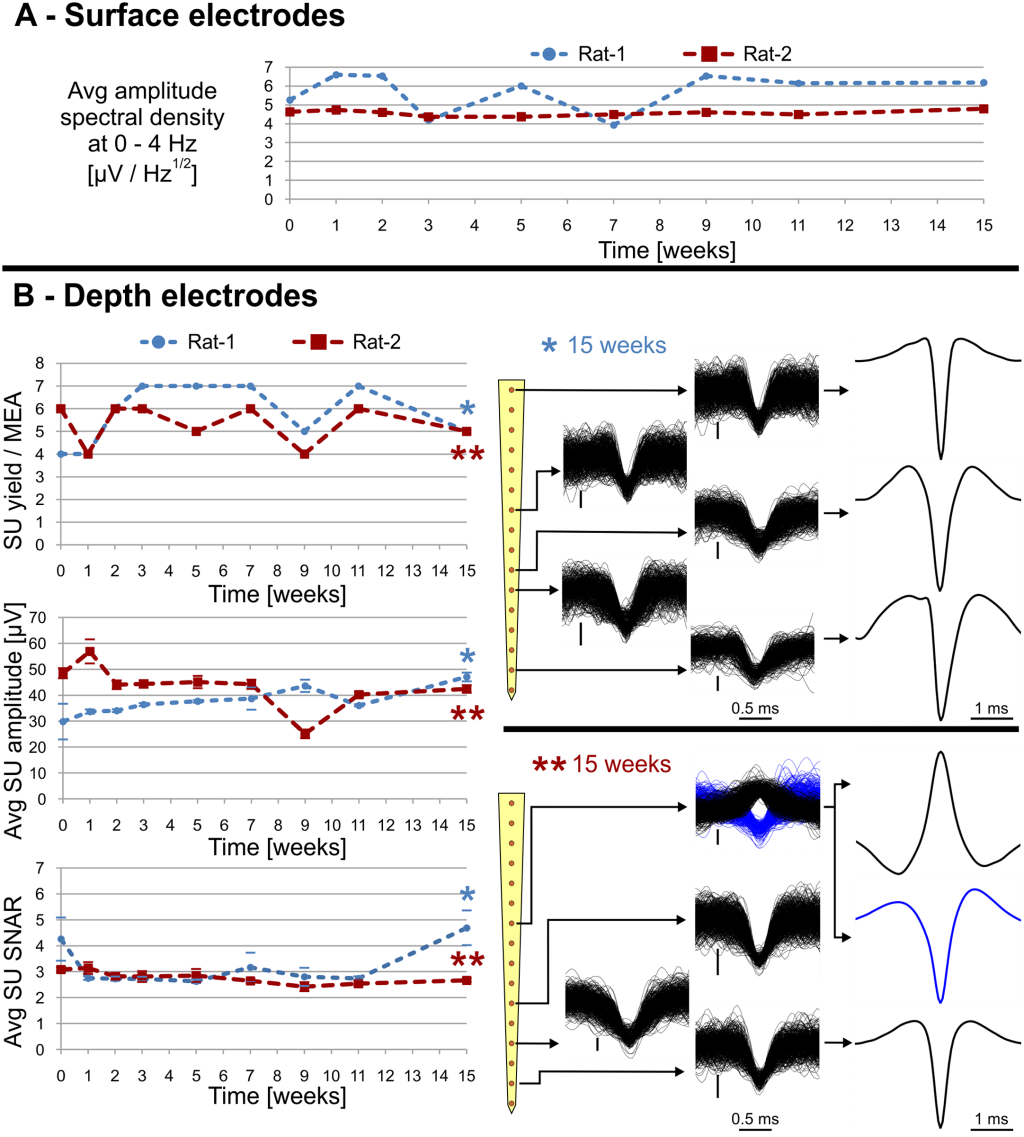
Chronic tests. (A) Variation of the average amplitude spectral density of the signals provided by surface electrodes in the 0–4 Hz band (characteristics of the sleeping state) (B) Variation of SU yield, average SU amplitude and average SU SNAR during 15 weeks, obtained from the chronically implanted rats. standard error to. Error bars indicate standard errors within a MEA. Piled single units waveforms, obtained from the last measurement of each rat are shown in the middle. Perpendicular scale bars are 20 μV. Examples of average single unit waveforms, corresponding to the different clusters are shown on the right.

Fig 8A and 8B, S2 Table and S1 Spike Data show the results of the chronic stability tests of the surface and depth measurements, respectively. We obtained recordings 15 weeks after probe implantation. The data at week 0 represent signals recorded 3–4 days after surgery. Changes occurred during the 15-week period, but there was no radical deterioration in the amplitude spectrum density of the signals provided by the surface electrodes. Regarding depth recordings, unit yield varied between 4–7 during the entire period, typical SU amplitudes are 30–50 μV. Average SU SNAR changed between 2.42 ± 0.35 and 4.69 ± 1.49. Interestingly, Rat-1 provided the best result on the 15^th^ week regarding SNAR. Comparing our results to the ones yielded by similar, polymer- [21] and silicon-based [48] linear probes, the SU yield and SNAR of our MEA are below average. Nevertheless, the graphs indicate stable performance and suitability for spike detection in the timescale of several months. The study is limited by the low number (2) of chronically examined animals and only shows that these probes can be capable of such performance, but gives no insight to the reproducibility of the measurements.

There are examples in the literature for the realization of ECoG measurements and simultaneous depth recordings underneath the ECoG-covered region with separate devices. MEMS surface MEAs with Parylene C—gold—Parylene C layer structure were used, along with tungsten fine wire microelectrodes, which could be inserted into the rat brain through holes on the surface arrays [49]. The configuration allowed LFP recordings with both the MEA and the fine wires, and the detection of unit activities with the latter. Surface grid arrays were synchronously used with the thumbtack laminar array and similar depth electrodes in humans with epilepsy [26, 50]. The results of our in vivo experiments indicate that these measurements can be realized with a single, all-flexible probe.

The application of polymer-based MEMS technology, along with microassembly allows high flexibility in the design of probe geometry, thus versatile three-dimensional sensor systems can be created. The number of electrodes on the ECoG and depth component, as well as their size and distance to each other can be adjusted in a wide range, tailored to different applications, and realized with high precision. There are no limitations against extending the depth component to a multiple shank MEA, neither in the microfabrication technology, nor in the assembly processes.

## Conclusions

To the authors’ knowledge, the device presented in this work is the first concerning polymer-based, flexible depth MEAs, combined with an array of also flexible brain surface electrodes. The applied microfabrication processes allowed us to precisely and reproducibly realize the designed probe with polyimide—platinum—SU-8 layer structure. The device is an upgraded version of the thumbtack-like fine wire electrode, which had been successfully used for obtaining depth recordings in the human neocortex. We characterized the new device in physiological saline, acutely and chronically in vivo in the central nervous system of rats, and showed its functionality. During in vivo recordings, electrodes both on the ECoG and on the extracellular component recorded LFP changes consistent with the expected waveforms and at the area of implantation. Furthermore, with the extracellular component, the detection of unit activities was possible for at least 15 weeks following the implantation. The applied rapid MEMS process flow along with a straightforward step of microassembly allows researchers to tailor three-dimensional probe geometries to various electrophysiological measurements.

## Supporting Information

S1 Acute Data The file contains data of acute recordings, including signals presented in Fig 7. Sampling rate: 20 kHz. It is an.avg file, compatible with the Neuroscan (Charlotte, NC, USA) software, compressed into a.zip file.
(ZIP)

S1 CSD Data A Matalab .m file, containing an avg matrix (which is the average of 10 periods of slow waves). The CSD can be calculated from the avg matrix and can be visualized using the CSDplotter toolbox with the following electrode positions: 0.0:0.2:3.0.
(MAT)

S1 Fig Polyimide microelectrode arrays on a 4-inch silicon wafer (JPG)

S1 Spike Data The file is a zipped folder, containing data of the spikes presented in Fig 8B. Matlab can be used in order to open the.fig files and to extract the data. E.g. the file Rat1_ClusterAvg02.fig represents the average single unit waveforms of the 2nd cluster of Rat-1. Piled single unit waveforms of Rat-2 measured on channel 7 are stored in Rat2_ch07.fig. The unit of the data values is 1 μV. Sampling rate: 20 kHz.
(ZIP)

S1 Table The results of electrochemical impedance spectroscopy measurements on a probe. (XLSX)

S2 Table The file is a Microsoft Excel document. Its first page contains the data obtained by depth electrodes (Fig 8B). Its second page contains data obtained by surface electrodes (Fig 8A).
(XLSX)
