# Supplementary figures and images for: A Multimodal, SU-8 - Platinum - Polyimide Microelectrode Array for Chronic In Vivo Neurophysiology
Source: PLoS One. 2015 Dec 18;10(12):e0145307. doi: 10.1371/journal.pone.0145307 (PMC4684315; doi:10.1371/journal.pone.0145307)

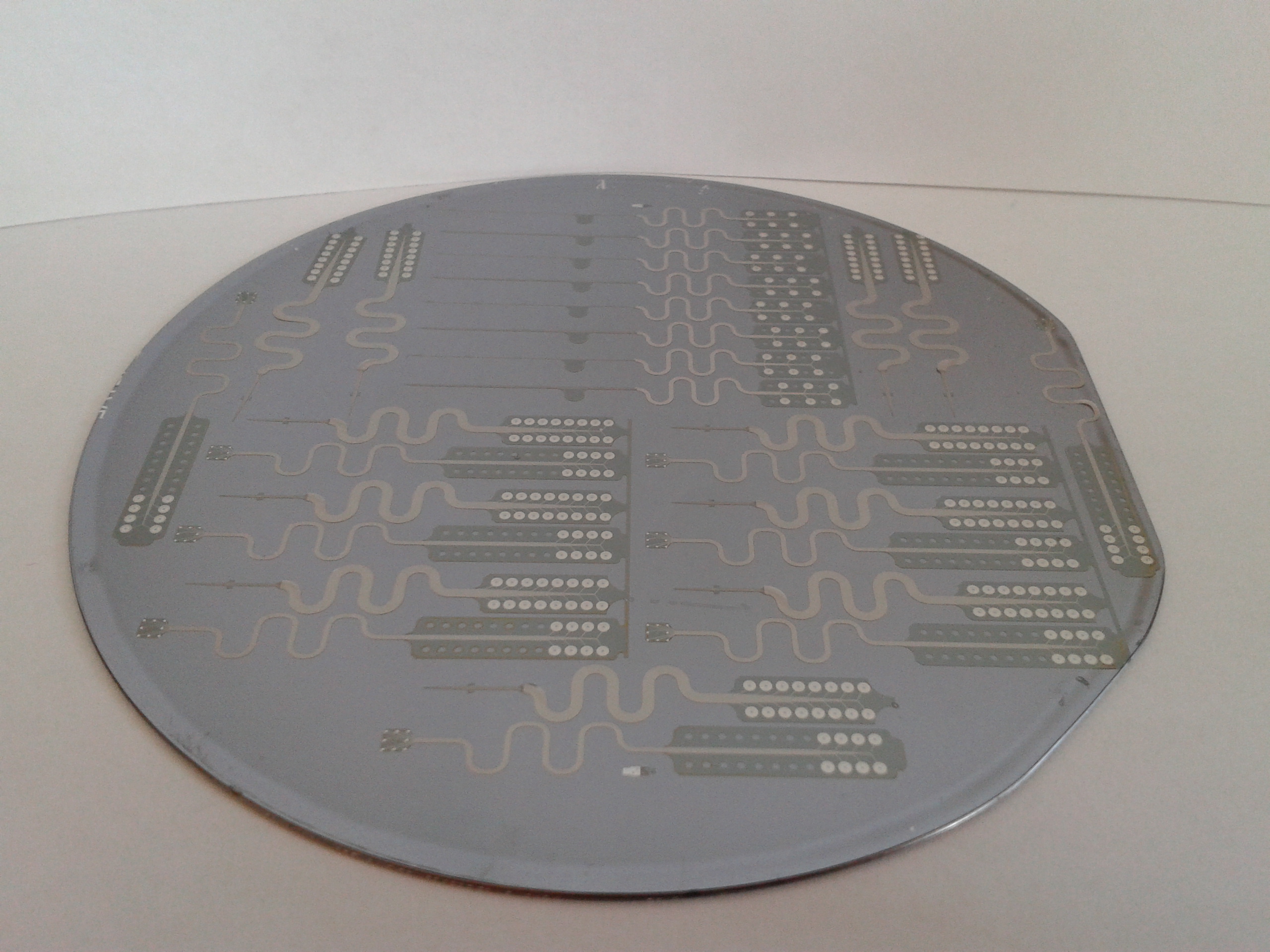

Supplement: S1 Fig — (JPG) [file pone.0145307.s003.jpg]
